# Water deficit modulates growth, secondary metabolism, and carvacrol biosynthesis gene expression in *Plectranthus amboinicus* (Lour.) Spreng

**DOI:** 10.1007/s12298-026-01737-z

**Published:** 2026-05-05

**Authors:** Abdel-Nasser El-Sheshtawy, Abeer Dahab, Hala Bayomy, Seham Almasoudi, Samah Youssef, Faisal Zulfiqar, Tamer Abd El-Ghany, Rasha El-Serafy, Nehad Elshayeb

**Affiliations:** 1 Environment and Bio-Agriculture Department, Faculty of Agriculture, Al-Azhar University, Cairo, 11884 Egypt; 2 Medicinal and Aromatic Plants Research Department, Agricultural Research Center, Horticulture Research Institute, Giza, 12619 Egypt; 3 Food Science and Nutrition Department, Faculty of Science, University of Tabuk, 71491 Tabuk, Saudi Arabia; 4 Horticulture Department, Faculty of Agriculture, Fayoum University, Fayoum, 63514 Egypt; 5 Department of Horticultural Sciences, Faculty of Agriculture and Environment, The Islamia University of Bahawalpur, Bahawalpur, 63100 Pakistan; 6 Korea University, Seoul, Republic of Korea; 7 Horticulture Department, Faculty of Agriculture, Tanta University, Tanta, 31527 Egypt; 8 Natural Resources Department, Faculty of African Postgraduate Studies, Cairo University, Giza, 12613 Egypt

**Keywords:** Secondary metabolites, Drought stress, Carvacrol, CYP71D178, CYP71D180, Essential oil, GC-mass

## Abstract

Plant secondary metabolites provide a unique basis for medications, flavorings, and industrial biochemicals. These metabolites frequently accumulate in plants exposed to environmental stressors (e.g., drought stress), as a mechanism for adaptation and resistance. *Plectranthus amboinicus* (Lour.) Spreng is a semi-succulent Lamiaceae species valued for its curative properties and essential oil, the concentration of which is heavily impacted by irrigation levels. This investigation reveals a novel study into the physiological and molecular mechanisms of *P. amboinicus* by correlating field capacity (FC) levels with metabolite accumulation and specific gene expression. We examined the impact of six irrigation levels (0, 20, 40, 60, 80 and 100%; denoted as FC-0 to FC-100) on growth, essential oil (EO) yield, and carvacrol biosynthesis during the 2022 and 2023 seasons. Data revealed that plants subjected to the FC-0 exhibited the lowest growth parameters. However, these plants also presented a higher essential oil percentage and carvacrol concentrations, in addition to an increase in polyphenols and antioxidant enzymes, with greater oxidative substances accumulation (H_2_O_2_ and MDA). Moreover, these plants presented higher gene expression of CYP71D178 and CYP71D180 genes. While increasing water level caused an increase in growth parameters, with a decline in the antioxidant activity. Increasing the water level to FC-100 increased the essential oil by approximately four fold but resulted in a decline in carvacrol content as compared to FC-0. Generally, water deficiency reduces crop yield, whereas irrigation increases plant productivity. However, in medicinal and aromatic plants, the relationship is distinct; in *Plectranthus amboinicus,* water deficiency showed a strong positive correlation with the secondary metabolite’s generation. In conclusion, the current report provides for the first time a molecular framework for *P. amboinicus* by depicting the expression patterns of CYP71D178 and CYP71D180. We revealed that these genes elaborate as the primary molecular shifts regulating carvacrol biosynthesis influenced by specific irrigation levels.

## Introduction

The World Health Organization reports that 80% of people worldwide rely on traditional herb-based therapies because they are inexpensive, accessible, and ofen have fewer adverse effects than allopathic drugs (Arumugam et al. 2016; Youssef et al. 2022). The existence of diverse, complex chemical-compounds- classified as secondary metabolites- is the reason for the curative qualities of these medicinal plants (Soni et al. 2015). These metabolites are also critical to the plant’s defense system, allowing it to withstand environmental stresses. Additionally, quantitative and qualitative changes in secondary metabolite biosynthesis occur when these plants are grown under stressors such as water deficiency (Pradhan et al. 2017).

*Plectranthus amboinicus* (Lour.) Spreng. is a semi-succulent shrub, that can climb or creep; reaching up to a meter in height (Roshan et al. 2010). It is a fleshy succulent plant with high aromatic properties, resembling oregano in flavor and smell. It grows as a perennial herb in the *Lamiaceae* family and is indigenous to tropical and warm regions (Arumugam et al. 2016). *P. amboinicus* is grown in gardens and containers because of its horticultural qualities and fragrance (Grayer et al. 2010). Its leaves are thick, pubescent, and broadly ovate (Roshan et al. 2010), and are consumed raw in traditional food as an ingredient and can be used as flavorings (Muniandy et al. 2014). *P. amboinicus* is one of the most significant aromatic medicinal plants due to its essential oil rich in bioactive synthesis. The herb produces an essential oil with high concentrations of carvacrol, β-caryophyllene, γ-terpinene, p-cymene, and α-terpineol (Murthy et al. 2009; Alshallash et al. 2022). These active ingredients display pharmacological properties such as antitumor, antioxidant, and antimicrobial properties (Murthy et al. 2009; Valera et al. 2003; Erny et al. 2014). *P. amboinicus* exhibits facultative Crassulacean Acid Metabolism (CAM), a pathway elicited in response to environmental stress-typically drought—which is downregulated once the stress ceases. Additionally, it possesses innate mechanisms to survive water scarcity, where prolonged stress triggers a complex defense response (Winter et al. 2020).

Drought impacts plant growth and development and is a challemge that must be addressed (Latif et al. 2022). It is considered the greatest environmental stress worldwide, particularly in arid and semi-arid regions. Plants growing in semi-arid regions suffer from drought stress due to water supply shortage accompanied with higher light intensities conditions (Kleinwächter and Selmar 2015). Spice plants grown in semi-arid regions, like the Mediterranean, typically have more aromatic compounds than their counterparts of the same species produced in temperate climates. Metabolic alterations caused by drought stress contribute to more active compounds and aroma in semi-arid areas (Singh-Sangwan et al. 1994). de Abreu and Mazzafera (2005) stated that medicinal plants grown in semi-arid regions typically have a higher concentration of the relevant natural compounds than those cultivated in a moderate Atlantic climate. Several environmental stressors can alter secondary metabolites concentrations and increases genes expression linked to the metabolite’s synthesis in medicinal plants (Selmar and Kleinwachter 2013). Drought stress modulates secondary metabolite synthesis, which is vital for plant defense and high-valuable active compounds accumulation (Emami Bistgani et al. 2024). Reactive oxygen species (ROS) accumulation under water-deficit conditions likely triggers improved metabolite production by altering plant cell signal transduction pathways (Khodabin et al. 2022).

Radwan et al. (2017) reported that varying degrees of water deficit affected the quantity and quality of sage essential oil. Additionally, they pointed to high correlation between the constituents percentage in the essential oil and the expression rate of genes synthesizing certain compounds. The effects of drought stress on two species of *Salvia nemorosa* L. and *Salvia reuterana* were evaluated by Bidabadi et al. (2020); their results revealed that 60% of field capacity revealed the maximum essential oil in both species. According to Ramezani et al. (2020) report, under water deficiency the concentrations of borneol, camphor, 1,8-cineole, and α- and β-thujone in sage increased. This increase was linked to the expression of genes present in their biosynthesis. Under water-deficit conditions, an increase in metabolite levels occurrs through the activation of stress pathways and shifting of metabolic priorities from primary growth to protective secondary metabolism (Selmar and Kleinwächter 2013).

Therefore, an in-depth study of the physiological behavior and stress response mechanisms of *P. amboinicus* is essential for understanding its drought tolerance. This study contributes to the functional validation of the CYP71D gene family (CYP71D178 and CYP71D180) as a molecular link between water availability and the quality of *P. amboinicus* essential oil. By linking transcriptomic responses with field capacity, we bridge the gap between the metabolic shift toward carvacrol production and water stress. The objective of this study was to evaluate variations in growth, herb yield, oil percentage, and the primary and secondary metabolite concentrations alongside the gene expression of carvacrol-related genes in *P. amboinicus* plants grown under different water deficit levels. The hypothesis was that drought-induced alterations in metabolite composition improve both the plant’s drought resilience and its pharmacological value.

## Materials and methods

### Characterization of the experimental site

Two pot investigations were conducted at the Experimental Farm of Environmental and Bio-Agriculture Department, Faculty of Agriculture, Al-Azhar University, Cairo, Egypt, (30°03*′*12′′ N and 31°19*′*05.2′′ E) during the period of February to September in 2022 and 2023. The data of climatic conditions for the experimental site during the experimental period are presented in Table 1. The chemical and physical characteristics of the studied soil are as follows: 59.58% sand, 15.31% silt, 25.11% clay (sandy clay loam texture), 66.10 mg kg^−1^ N, 7.85 mg kg^−1^ P, and 280.30 mg kg^−1^ K with pH = 8 and EC = 0.85 ds cm^−1^.

**Table 1:** Temperature (°C), wind speed (m s^−1^), relative humidity (%), average precipitation (mm day^−1^) and surface pressure (kPa) of experimental site during seasons 2022 and 2023

Month	Temperature (°C)		Wind speed (m s^–1^)		Relative Humidity (%)	Average Precipitation (mm day^–1^)	Surface pressure (kPa)
	Max	Min	Max	Min			
2022							
Feb	19.48	6.57	5.67	2.20	66.60	0.39	100.22
Mar	21.18	7.03	6.66	2.33	59.98	1.30	100.21
Apr	31.43	13.51	6.51	2.69	44.97	0.00	99.61
May	33.65	16.68	6.99	2.52	39.75	0.00	99.74
Jun	37.44	20.94	7.13	2.07	42.11	0.00	99.35
Jul	38.36	21.41	6.85	2.12	42.45	0.06	99.15
Aug	38.45	22.73	6.62	1.90	46.09	0.06	99.20
2023							
Feb	20.48	8.27	4.94	2.15	68.53	2.60	100.24
Mar	18.88	6.38	5.20	2.09	69.79	0.38	100.40
Apr	25.65	10.82	6.20	1.83	54.11	0.19	99.79
May	29.59	13.35	6.34	2.02	45.51	0.10	99.67
Jun	33.53	16.98	7.38	2.14	42.51	0.03	99.56
Jul	37.66	21.39	7.64	2.12	39.58	0.04	99.39
Aug	40.51	22.74	7.27	2.22	40.54	0.00	99.22

### Planting methodology

Uniform *P. amboinicus* cuttings (15 cm) were taken from mother plants (two years-old plants) grown in the same Experimental Farm and each three cuttings were cultivated in 30 cm pots filled with soil on the 1st of February for both seasons. After four weeks (root establishment), the pots were divided into six groups and were subjected for 170 days (March-August) to the following treatments: 100% of field capacity (FC) as control plants (FC-100), 80% of FC (FC-80), 60% of FC (FC-60), 40% of FC (FC-40), 20% of FC (FC-20), and 0% FC (unwater plants) (FC-0). Treatments were arranged in a complete randomized design (CRD) with 3 replicates for each group, each group included six pots. The FC of experimental soil was assessed using the method of Bouyoucos (1983). The field capacity (FC%) determined in the 100, 80, 60, 40, and 20% FC treatments were 23.2, 18.56, 13.92, 9.28, and 6.64%, respectively. For maintaining soil water content at the specific FC, each pot was daily weighed and the amount of lost water was compensated (Beadle et al. 1985).

### Plant architecture and biomass yield

The plants were collected for growth analysis after 170 days of cultivation. All plants from each treatment were separately collected and the plant height, number of branches and leaves, leaf area, and the herb, leaves, and stem fresh weight were estimated. Then the shoots were air dried for a week, and oven dried at 70 ºCtill constant weights were recorded.

### Relative water content and membrane stability index

Relative water content (RWC) was determined using the methods of Barrs (1968) using the following formulas: RWC = (FW – DW) ÷ (TW – DW) × 100. Membrane stability index (MSI) was determined according to Sairam et al. (1997) using the following equation: MSI = [1-(C1/C2)] × 100.

### Essential oil extraction and GC–MS analysis

The essential oil in the fresh herb was extracted by the water distillation method of Viuda-Martos et al. (2011) for 3 h using the Clevenger apparatus for oil percent and oil yield determination. The fresh herb (100 g) from each treatment was distilled in triplicate and the oil content is presented as the average value. The extracted oil was dried using anhydrous sodium sulfate. Identifying the active substances of the essential oil was performed by a Perkin Elmer GC-MS analysis (model: Clarus 580/560 S) equipped with four capillary columns (30 m X 0.25 mm ID and film thickness 0.25 μm). Essential oil components were identified by comparing their retention times and mass spectrum with those of standards, NIST library of the GC-MS system and literature data.

### Physiological and biochemical analysis

For biochemical analysis, 170 days after cultivation, leaf samples were taken and immediately kept in liquid nitrogen, then using a mortar, it was pulverized to a fine powder and kept at − 80 °C.

#### Photosynthetic pigments

Chlorophyll a, b, and carotenoids content were measured spectrophotometrically (6800 UV/Vis Spectrophotometer, Jenway, Bibby Scientific Ltd., Staffordshire, UK) using acetone following the method of Mitic et al. (2013) at wavelengths of 662, 644, and 440 nm. Pigments concentrations were calculated using the formula of Holm (1954) and Von Wettstein (1957).

#### Quantification of total phenolic content and antioxidant activity

Total phenolic content was quantified in dried leaves extracts using methanol. The protocol of Dewanto et al. (2002) was followed for phenolics estimation and gallic acid was used as a standard. The calorimetric procedure for quantifying Ferric Ion Reducing Power Assay (FRAP) using Benzie and Strain’s (1996) methods was followed.

#### Activities of antioxidant enzymes

Antioxidant enzyme activities were assessed using a spectrophotometric procedure. A 0.5 g of frozen leaf tissue was homogenized in 5 mL of 50 mM potassium phosphate buffer (pH 7.8 for SOD and pH 7.0 for CAT), 1% (w/v) polyvinylpyrrolidone (PVP), and 0.1 mM EDTA. Then the homogenate was centrifuged at 4 °C for 20 min at 12,000 × g, and the obtained supernatant was utilized for Superoxide Dismutase (SOD) and Catalase (CAT) determination. SOD (EC 1.15.1.1) measuring was done according to Giannopolitis and Ries (1977); while CAT (EC 1.11.1.6), estimation was carried out according to the method described by Aebi (1984).

#### ROS accumulation

Malondialdehyde (MDA) was estimated in the frozen leaf samples following the method of Heath and Packer (1968), while hydrogen peroxide (H_2_O_2_) was quantified using the protocol of Patterson et al. (1984).

#### RNA extraction and cDNA Synthesis

The total RNA of *P. amboinicus* leaves was isolated by RNAXTM_PLUS Kit (SinaClon Bioscience Co., Karaj, Iran) according to manufacturer’s instructions and cDNA synthesized using M-MuLV reverse transcriptase based on the method, as reported by previously (Elyasi et al. 2016).

#### qRT-PCR and gene expression analysis

Gene expression was analyzed using three biological replicates and three technical replications per replicate. The particular primers for qRT-PCR were constructed based on the previously acquired conserved sequencing results of Crocoll et al. (2010) and Tohidi et al. (2020), with ubiquitin serving as a reference gene for normalization. The qRT-PCR settings were as follows: initial denaturation at 94 ^◦^C for 40 s, followed by 40 cycles of denaturation at 94 ^◦^C for 5 s, annealing at 52–62 ^⸰^C for 35 s, and elongation at 72 ^◦^C for 25 s. Annealing temperatures were 54 ^◦^C for the ubiquitin primer and 60 ^◦^C for the CYP71D178 and CYP71D180 primers (Table 2). Normalized raw Ct values were used to compare the results of control and treated samples using the 2^−∆∆Ct^ (fold change) method, as described previously by Livak and Schmittgen (2001).

**Table 2:** Primers used to qRT-PCR analysis

Real-Time Primers	Sequences (5′ to 3′)	Tm °C	Amplicon Size (bp)
CYP71D178 F CYP71D178 R	CGACCACTGGCGCCAAATAC CGATGGTGCACACCGTCTTG	60	179
CYP71D180 F CYP71D178 R	AACATCGGGTCGCGGATCAT GATGAGGCGAGCCATCTCGT	60	165
UQ F UQ R	AAGACCTACACCAAGCCCAA AAGTGAGCCCACACTTACCA	54	196

### Statistical analysis

In this study, a randomized complete design was used as an experimental layout. To compare the significant differences between treatments, the analysis of variance (ANOVA) using COSTAT was made for the data obtained according to Snedecor and Cochran (49). Tukey’s test was used for post-hoc analysis (*p* < 0.05).

## Results

### Plant architecture and biomass yield

**Fig. 1:**
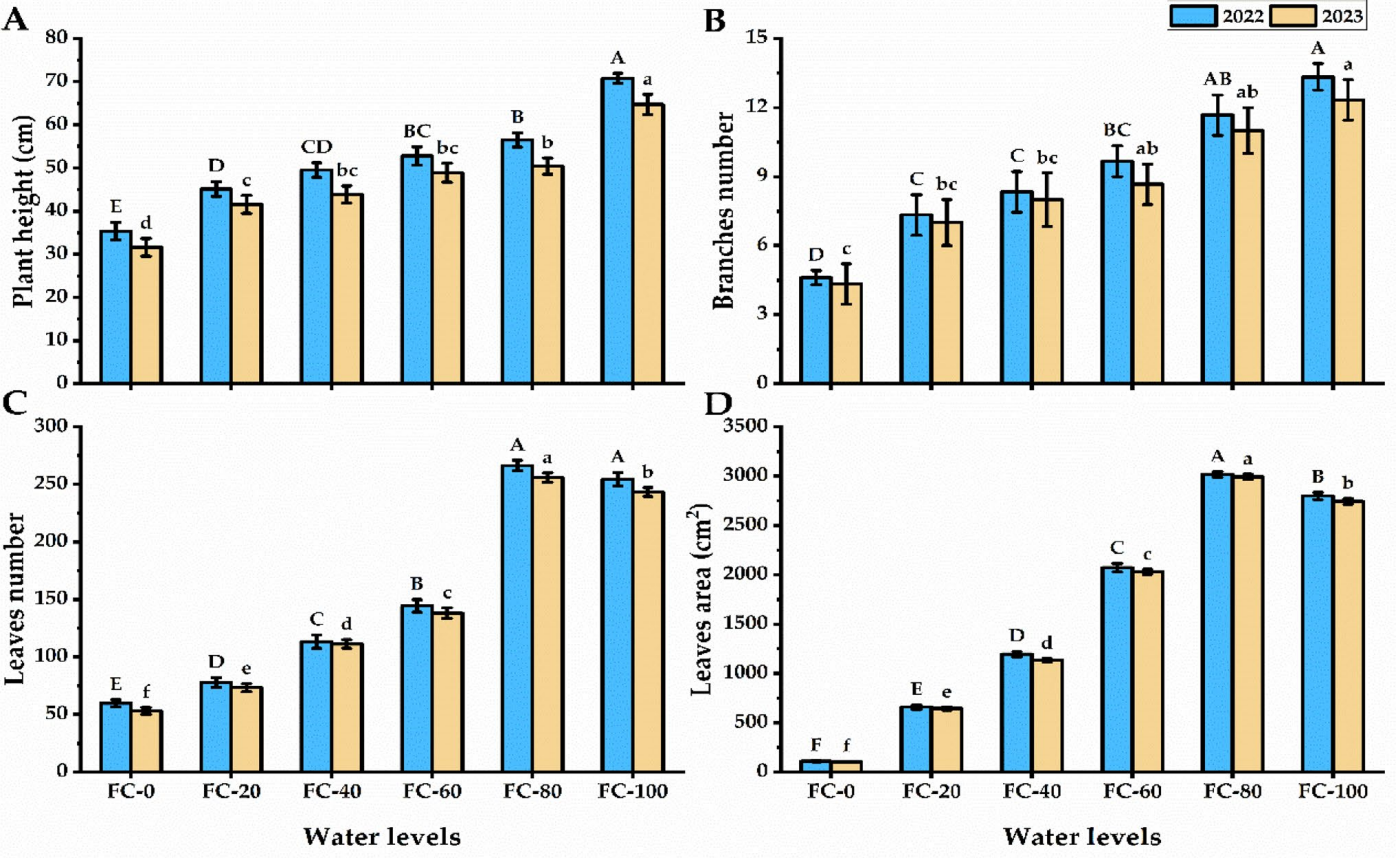
Plant height (**A**), branches number (**B**), leaves number (**C**), and leaves area (**D**) of *Plectranthus amboinicu*s (Lour.) Spreng. plant in response to different water levels. Data are mean value ± SE. Bars with same letters are not significant at *p* ≤ 0.05 level. FC-0, FC-20, FC-40, FC-60, FC-80, and FC-100 are the water levels at 0, 20, 40, 60, 80, and 100% of field capacity, respectively

**Fig. 2:**
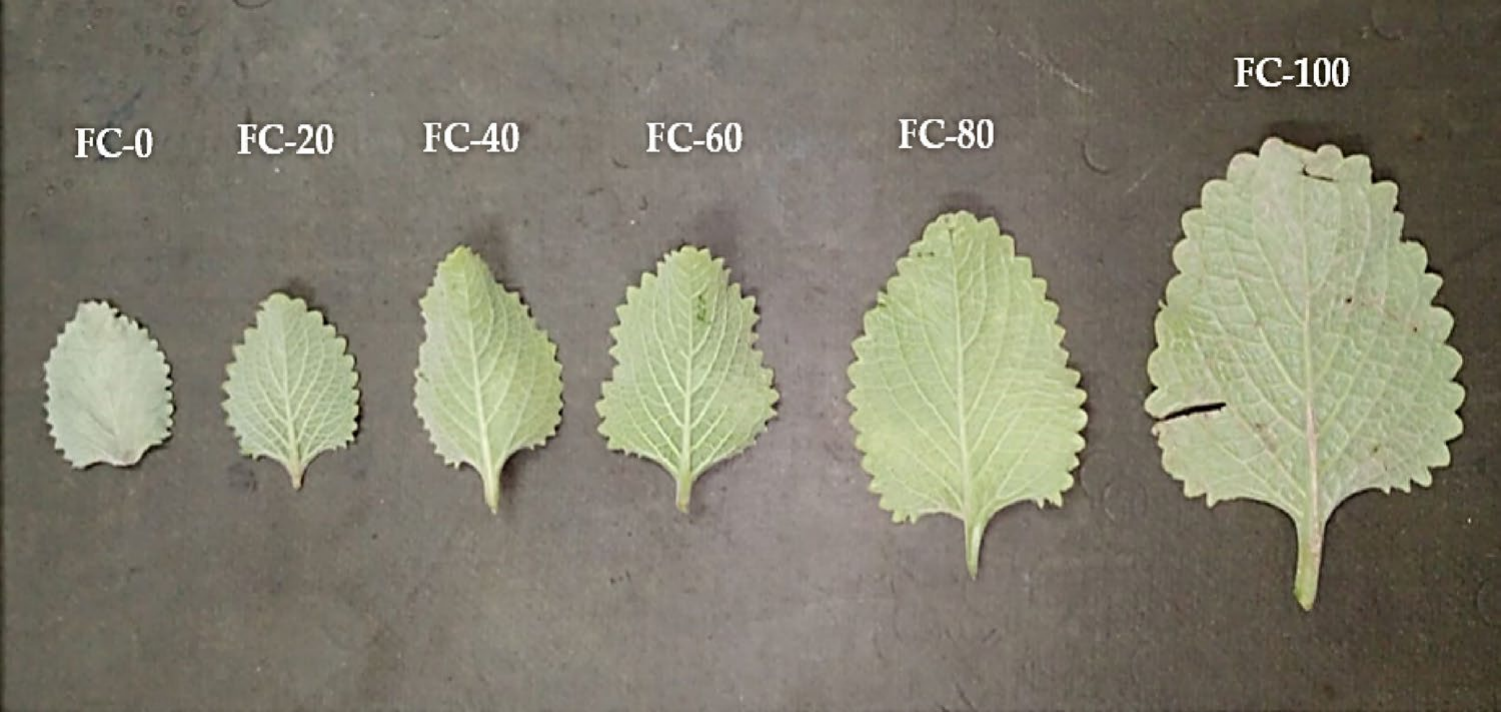
Phenotypic changes in *Plectranthus amboinicu*s (Lour.) Spreng. leaves in response to different water levels of field capacity

Drought stress presented a significant decline in the growth and biomass yield of *P. amboinicus* plants but increasing water level caused a slight stimulation in their growth and biomass values (Figs. 1 and 2). The plants subjected to 100% water level presented the tallest plants with a significant enhancement relative to FC-0 plants alongside with more branches number which carried more leaves. A significant increase in the leaves number in FC-80 and FC-100 plants were noticed as compared with FC-0 plants for seasons. The growth in leaves’ number is followed by significant growth in their leaf area. On the other hand, the shortest plants were exhibited by FC-0 plants which carried lower branches and leaves number with lower leaf area.

Concerning the herb fresh biomass, the lowest values of leaves and stem fresh weights as well as herb biomass were exhibited by *P. amboinicus* plants subjected to FC-0 treatment, and these values were increased gradually with elevating water level to reach the maximum values by FC-100 plants. The highest dry biomass for leaves, stems, and herbs were detected in FC-80 plants with significant differences with the other water levels for both seasons.

### RWC and MSI

**Fig. 3:**
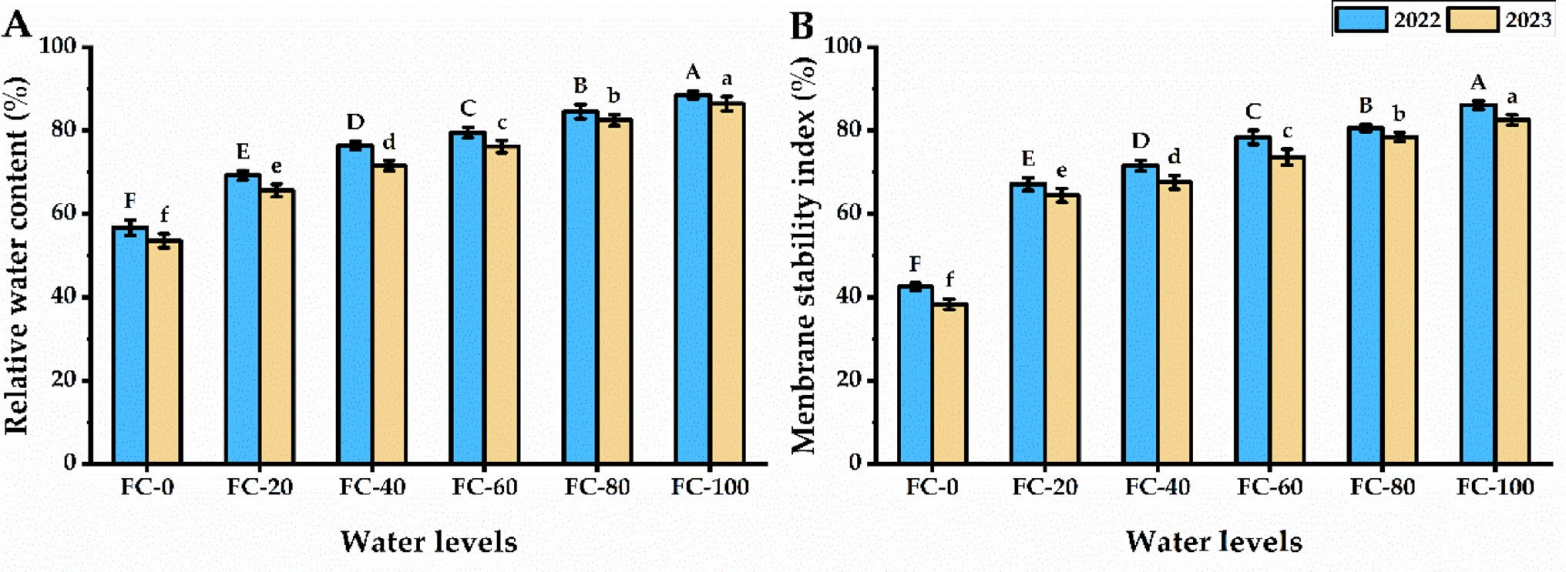
Relative water content (**A**) and membrane stability index (**B**) of *Plectranthus amboinicu*s (Lour.) Spreng. plant in response to different water levels. Data are mean value ± SE. Bars with same letters are not significant at *p* ≤ 0.05 level. FC-0, FC-20, FC-40, FC-60, FC-80, and FC-100 are the water levels at 0, 20, 40, 60, 80, and 100% of field capacity, respectively

Leaves RWC of *P. amboinicus* plants showed strong response to water deficiency levels (Fig. 3). A significant growth was observed with elevating the water level to FC-100 that maximized RWC value by about 56.3 and 61.6% than FC-0 level for both seasons, respectively.

Water deficiency negatively affected the MSI of *P. amboinicus* leaves. The lowest MSI was observed by plants exposed to FC-0 treatment but increasing the water level led to an improvement in the MSI value to reach 86.1 and 82.6% for the first and second seasons, respectively.

### Essential oil yield

**Fig. 4:**
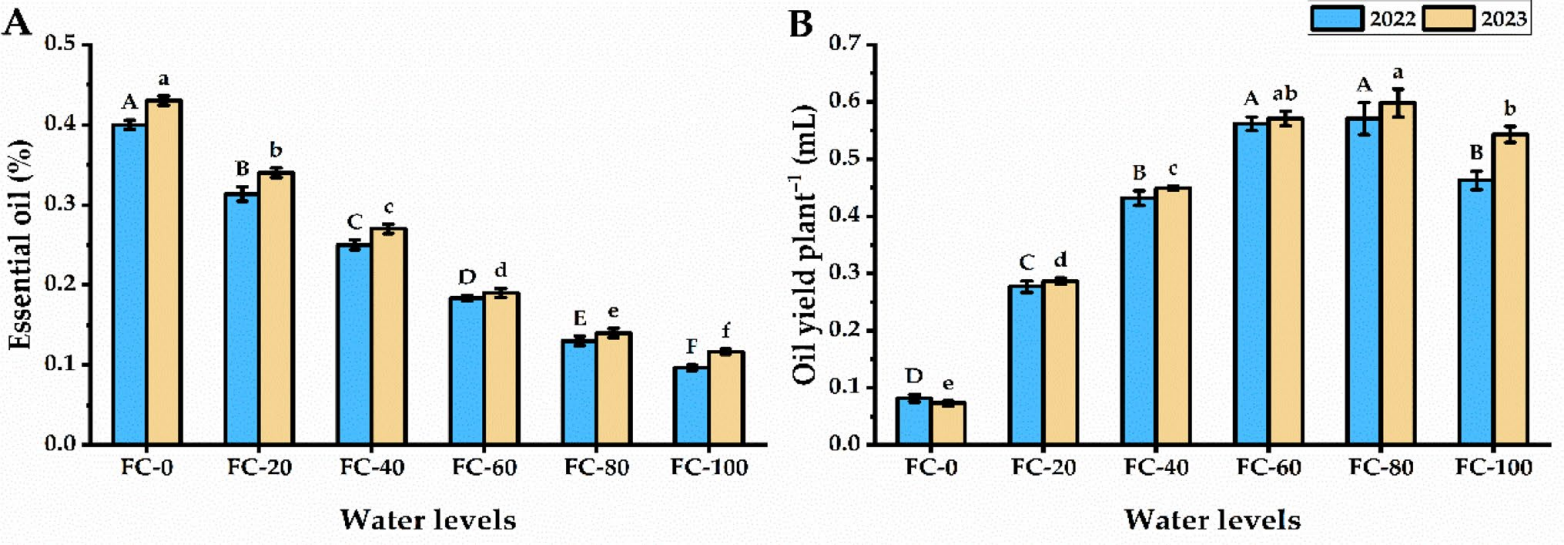
Essential oil percentage (**A**) and oil yield per plant (mL) (**B**) of *Plectranthus amboinicu*s (Lour.) Spreng. plant in response to different water levels. Data are mean value ± SE. Bars with same letters are not significant at *p* ≤ 0.05 level. FC-0, FC-20, FC-40, FC-60, FC-80, and FC-100 are the water levels at 0, 20, 40, 60, 80, and 100% of field capacity, respectively

Essential oil percent was slightly affected by water deficiency, as subjected plants to FC-0 exhibited the maximum oil % (0.40 and 0.43% for the first and second seasons, respectively), while increasing water level led to a significant decrease in the essential oil % to reach the lowest value when *P. amboinicus* plants subjected to FC-100 treatment in both seasons (Fig. 4). Plants subjected to water deficiency significantly showed an increase in their corresponding oil yield with increasing the amount of supplemented water to record the highest essential oil yield by FC-100 plants.

### Essential oil composition

Drought stress exhibited great variations in the secondary metabolite’s concentration in the *P. amboinicus* essential oil (Table 3). Carvacrol, α-Terpinolene, α-Amorphene, β-Cymene, p-Mentha-1, copaene, Caryophyllene, beta cubebene, tau-Cadinol are the major components obtained by GC-Mass analysis. Carvacrol is the main component in the essential oil of *P. amboinicus* herb, which showed a strong and significant increase by about 2.9-fold when decreasing the water level from FC-100 to FC-0. The same trend was noticed by α-Terpinolene, β-Cymene, p-Mentha-1, Alpha-thujene, Linalool components. On contrast to the behavior of the previous components, increasing the water level led to a gradual increase in the α-Amorphene, α-Terpinolene, β-Cymene, p-Mentha-1, Caryophyllene, à-Caryophyllene, beta cubebene, δ-Cadinene, tau-Cadinol, Neoisolongifolene, 8,9-dehydro- components to reach the highest values at the highest water levels of FC-80 and FC-100.

**Table 3:** GC-Mass analysis of *Plectranthus amboinicu*s (Lour.) Spreng. plant in response to different water levels

Compound	Approximate RT	Treatments					
		FC-0	FC-20	FC-40	FC-60	FC-80	FC-100
1-Octen-3-ol	7.23	1.10 ± 0.05	0.51 ± 0.004	0.47 ± 0.005	0.43 ± 0.006	0.17 ± 0.004	–
α-Terpinolene	8.09	3.99 ± 0.07	3.82 ± 0.07	2.14 ± 0.02	1.96 ± 0.02	2.65 ± 0.06	2.34 ± 0.03
β-Cymene	8.30	8.32 ± 0.07	3.59 ± 0.69	2.86 ± 0.03	2.98 ± 0.02	3.33 ± 0.05	3.06 ± 0.05
p-Mentha-1	8.97	6.81 ± 0.11	5.42 ± 0.02	4.60 ± 0.01	4.44 ± 0.03	4.53 ± 0.08	4.29 ± 0.03
Alpha-thujene	9.47	2.37 ± 0.08	1.04 ± 0.04	0.89 ± 0.003	0.92 ± 0.005	0.65 ± 0.005	0.64 ± 0.002
Linalool	9.64	2.46 ± 0.08	0.95 ± 0.01	0.88 ± 0.01	0.81 ± 0.004	0.46 ± 0.01	0.49 ± 0.003
Alpha-terpineol	11.05	1.33 ± 0.03	0.66 ± 0.04	0.47 ± 0.04	0.41 ± 0.01	0.29 ± 0.01	0.30 ± 0.01
δ-2-Carene	11.95	1.02 ± 0.02	–	–	–	–	–
Carvacrol	13.08	16.56 ± 0.04	8.36 ± 0.02	8.06 ± 0.03	7.66 ± 0.02	6.29 ± 0.13	5.76 ± 0.03
Copaene	14.25	2.76 ± 0.03	4.42 ± 0.04	4.08 ± 0.02	3.97 ± 0.01	4.57 ± 0.16	4.63 ± 0.02
Beta elemene	14.42	–	1.73 ± 0.05	1.48 ± 0.01	1.43 ± 0.01	1.54 ± 0.01	1.82 ± 0.03
Caryophyllene	15.20	4.55 ± 0.03	6.70 ± 0.02	7.11 ± 0.03	7.81 ± 0.04	6.79 ± 0.08	7.03 ± 0.03
à-Caryophyllene	15.95	4.45 ± 0.02	6.63 ± 0.05	7.44 ± 0.03	7.69 ± 0.01	7.13 ± 0.10	7.35 ± 0.06
beta cubebene	16.53	5.37 ± 0.03	7.26 ± 0.01	7.56 ± 0.03	7.68 ± 0.01	9.49 ± 0.08	9.27 ± 0.04
γ-Gurjunene	16.68	1.12 ± 0.05	0.48 ± 0.01	0.47 ± 0.01	0.52 ± 0.003	0.44 ± 0.003	0.78 ± 0.005
δ-Cadinene	16.66	0.29 ± 0.004	2.22 ± 0.01	2.43 ± 0.02	2.52 ± 0.004	2.36 ± 0.03	2.33 ± 0.02
Amorphene	17.27	9.22 ± 0.09	8.57 ± 0.03	9.37 ± 0.02	9.70 ± 0.05	10.23 ± 0.07	9.92 ± 0.03
Spathulenol	18.34	1.16 ± 0.02	2.45 ± 0.02	2.39 ± 0.03	2.07 ± 0.03	1.91 ± 0.005	2.39 ± 0.03
tau-Cadinol	19.84	9.37 ± 0.08	10.09 ± 0.08	11.47 ± 0.01	11.01 ± 0.08	12.49 ± 0.02	12.86 ± 0.07
alpha cadinol	20.12	–	5.96 ± 0.03	7.15 ± 0.02	–	–	–
Carotol	20.86	–	5.35 ± 0.03	5.18 ± 0.06	4.82 ± 0.10	4.21 ± 0.03	4.78 ± 0.03
Isolongifolen-9-one	21.18	1.14 ± 0.005	1.16 ± 0.02	1.11 ± 0.01	1.58 ± 0.02	1.36 ± 0.02	1.25 ± 0.02
Neoisolongifolene, 8,9-dehydro-	22.08	1.13 ± 0.02	2.65 ± 0.05	2.67 ± 0.02	2.76 ± 0.06	2.92 ± 0.01	3.06 ± 0.03

FC-0, FC-20, FC-40, FC-60, FC-80, and FC-100 are the water levels at 0, 20, 40, 60, 80, and 100% of field capacity, respectively. Mean values sharing the same letter in the same column are not differ significantly at p ≤ 0.05 by Tukey

### Photosynthetic pigments

*P. amboinicus* plants exposed to water deficiency showed a significant decline in their leaf pigments concentration (a, b, carotenoid, and total chlorophyll) (Table 4). The lowest total chlorophyll (1.27 and 1.31 mg g^-1^ FW for the first and second seasons, respectively) and carotenoids content (0.49 and 0.52 mg g^-1^ FW for the first and second seasons, respectively) were observed by FC-0 plants, and these values were increased to reach the highest in the leaves of FC-100 plants which recorded 2.30 and 2.36 mg g^-1^ FW for total chlorophyll, and 0.83 and 0.86 mg g^-1^ FW for carotenoids content for both seasons, respectively.

**Table 4:** Leaf pigments of *Plectranthus amboinicu*s (Lour.) Spreng. plant in response to different water levels for two seasons of 2022 and 2023

Treatments	Chlorophyll a (mg g^−1^ FW)		Chlorophyll b (mg g^−1^ FW)		Carotenoids (mg g^−1^ FW)		Total chlorophyll (mg g^−1^ FW)	
	2022	2023	2022	2023	2022	2023	2022	2023
FC-0	0.81 ± 0.01 d	0.83 ± 0.01 e	0.046 ± 0.01 e	0.49 ± 0.03 e	0.49 ± 0.01 d	0.52 ± 0.01 c	1.27 ± 0.02 e	1.31 ± 0.03 f
FC-20	1.19 ± 0.01 c	1.24 ± 0.01 d	0.57 ± 0.02 d	0.63 ± 0.02 d	0.55 ± 0.01 c	0.58 ± 0.01 c	1.75 ± 0.03 d	1.87 ± 0.02 e
FC-40	1.22 ± 0.05 c	1.26 ± 0.02 d	0.71 ± 0.02 c	0.76 ± 0.01 c	0.71 ± 0.02 b	0.74 ± 0.03 b	1.93 ± 0.06 c	2.01 ± 0.03 d
FC-60	1.37 ± 0.03 b	1.40 ± 0.02 c	0.81 ± 0.03 b	0.85 ± 0.02 b	0.76 ± 0.03 ab	0.78 ± 0.02 b	2.18 ± 0.07 b	2.25 ± 0.03 c
FC-80	1.63 ± 0.03 a	1.65 ± 0.01 a	0.93 ± 0.012 a	0.98 ± 0.02 a	0.83 ± 0.01 a	0.86 ± 0.03 a	2.56 ± 0.03 a	2.63 ± 0.03 a
FC-100	1.46 ± 0.01 b	1.49 ± 0.02 b	0.84 ± 0.01 b	0.87 ± 0.01 b	0.78 ± 0.03 ab	0.80 ± 0.02 b	2.30 ± 0.02 b	2.36 ± 0.01 b

FC-0, FC-20, FC-40, FC-60, FC-80, and FC-100 are the water levels at 0, 20, 40, 60, 80, and 100% of field capacity, respectively. Mean values sharing the same letter in the same column are not differ significantly at *p* ≤ 0.05 by Tukey

### Polyphenols and FRAP assay

Leaves polyphenols content in *P. amboinicus* subjected to FC-0 showed a significant increase in comparison to the other treatments (Table 5), this value gradually decreased with increasing the supplemented water level. The highest polyphenols level was recorded by FC-0 plants with an increase about 1.2 and 1.4-fold than FC-100 plants for the first and second seasons, respectively. But the lowest values were obtained by FC-80 treated plants. On contrary to total polyphenols behavior, FRAP content exhibited that increasing supplemented water to 80% led to an increase by about 0.7-fold than FC-0 plants for both seasons, but increasing the supplemented water to 100% of field capacity showed a reduction in FRAP content after that.

**Table 5:** Polyphenols, Ferric Reducing Antioxidant Power (FRAP), catalase (CAT), and Superoxide Dismutase (SOD) enzymes of *Plectranthus amboinicu*s (Lour.) Spreng. plant in response to different water levels for two seasons of 2022 and 2023

Treatments	Polyphenols mg g^−1^		FRAP mg g^−1^		CAT U mg^−1^		SOD mg g^−1^	
	2022	2023	2022	2023	2022	2023	2022	2023
FC-0	3.7 ± 0.2 a	3.89 ± 0.1 a	0.15 ± 0.004 f	0.19 ± 0.01 f	14.60 ± 0.57 a	15.26 ± 0.59 a	2.52 ± 0.08 a	2.83 ± 0.1 a
FC-20	3.09 ± 0.01 b	3.22 ± 0.1 b	0.32 ± 0.01 e	0.39 ± 0.01 e	11.93 ± 0.51 b	12.27 ± 0.67 b	2.14 ± 0.07 b	2.39 ± 0.1 b
FC-40	2.61 ± 0.01 c	2.66 ± 0.2 c	0.43 ± 0.03 d	0.54 ± 0.02 d	10.31 ± 0.58 c	10.97 ± 0.51 b	1.93 ± 0.06 c	2.17 ± 0.1 cd
FC-60	2.24 ± 0.01 d	2.58 ± 0.2 cd	0.69 ± 0.04 c	0.72 ± 0.01 c	8.85 ± 0.35 c	9.10 ± 0.43 c	1.81 ± 0.06 cd	1.90 ± 0.1 cd
FC-80	1.91 ± 0.07 f	2.06 ± 0.2 d	0.85 ± 0.02 a	0.91 ± 0.02 a	4.54 ± 0.17 d	5.01 ± 0.26 d	1.39 ± 0.04 e	1.51 ± 0.03 e
FC-100	2.48 ± 0.01 e	2.50 ± 0.21 e	0.76 ± 0.02 b	0.78 ± 0.03 b	9.01 ± 0.38 c	9.46 ± 0.30 c	1.63 ± 0.05 d	1.73 ± 0.1 de

FC-0, FC-20, FC-40, FC-60, FC-80, and FC-100 are the water levels at 0, 20, 40, 60, 80, and 100% of field capacity, respectively. Mean values sharing the same letter in the same column are not differ significantly at *p* ≤ 0.05 by Tukey

### Antioxidants enzymes

Date presented in Table 5 revealed that the highest activities of CAT and SOD enzymes were obtained by the lowest water levels relative to higher water levels, and a decline in their activities were noticed with increasing water levels to reach the lowest in the leaves of FC-80 plants.

### MDA and H_2_O_2_ content

Increasing the water level supplemented to *P. amboinicus* plants caused a decline in their leaves MDA and H_2_O_2_ concentrations (Table 6). The highest MDA and H_2_O_2_ levels were observed in FC-0 leaves and these values were decreased gradually to reach the minimum values by FC-80 plants as recorded 1.38 and 1.41 mmol g^−1^ for MDA and 14.95 and 13.21 μg g^−1^ FW for H_2_O_2_ for the first and second seasons, respectively but these values were increased after that with increasing water level to 100%.

**Table 6:** Malondialdehyde (MDA) and hydrogen peroxide (H_2_O_2_) levels in *Plectranthus amboinicu*s (Lour.) Spreng. leaves in response to different water levels for two seasons of 2022 and 2023

Treatments	MDA (mmol g^−1^)		H_2_O_2_ (μg g^−1^ FW)	
	2022	2023	2022	2023
FC-0	1.64 ± 0.006 a	1.72 ± 0.02 a	28.6 ± 0.04 a	30.2 ± 0.32 a
FC-20	1.61 ± 0.01 a	1.66 ± 0.02 b	25.1 ± 0.16 b	22.1 ± 0.26 b
FC-40	1.51 ± 0.01 b	1.61 ± 0.01 c	23.2 ± 0.25 c	20.2 ± 0.32 c
FC-60	1.44 ± 0.01 c	1.56 ± 0.01 d	18.9 ± 0.28 d	16.9 ± 0.35 d
FC-80	1.38 ± 0.03 d	1.41 ± 0.02 e	14.9 ± 0.19 f	13.2 ± 0.23 f
FC-100	1.40 ± 0.02 cd	1.43 ± 0.01 e	16.8 ± 0.24 e	15.2 ± 0.27 e

FC-0, FC-20, FC-40, FC-60, FC-80, and FC-100 are the water levels at 0, 20, 40, 60, 80, and 100% of field capacity, respectively. Mean values sharing the same letter in the same column are not differ significantly at *p* ≤ 0.05 by Tukey

### Gene expression

**Fig. 5:**
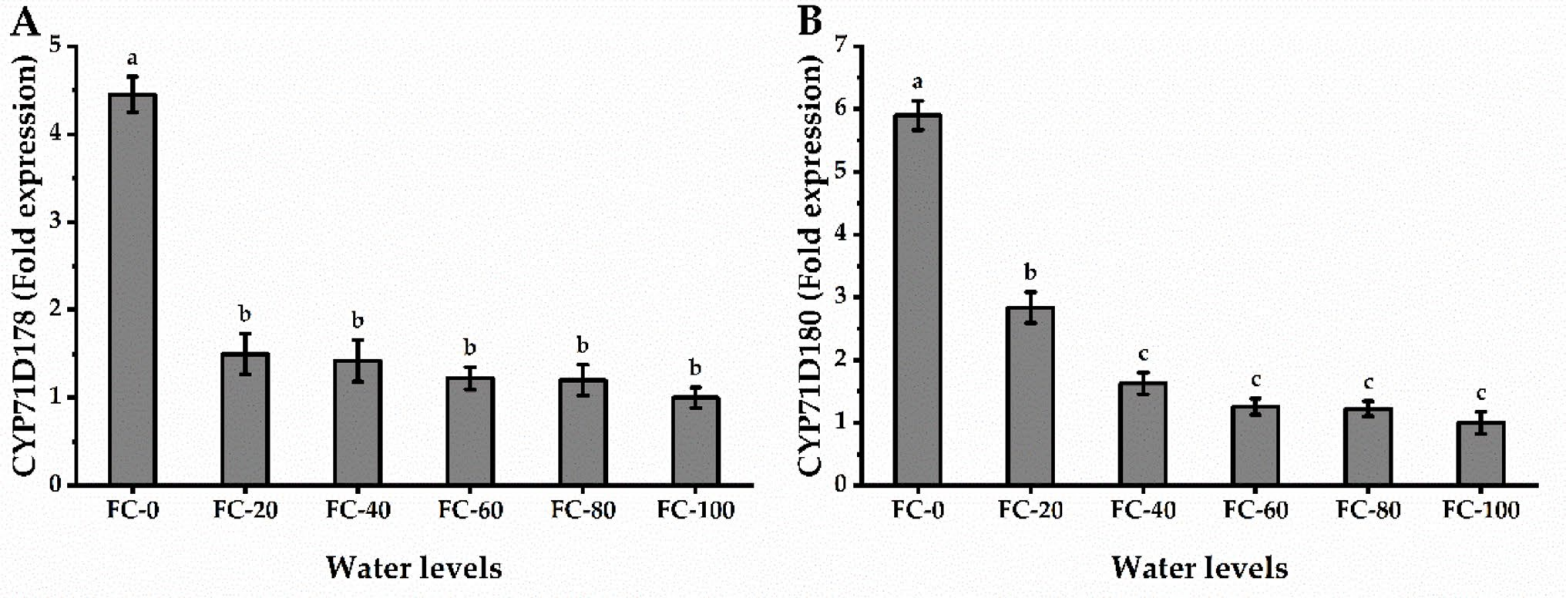
Gene relative expression of CYP71D178 (**A**) and CYP71D180 (**B**) of *Plectranthus amboinicu*s (Lour.) Spreng. plant in response to different water levels. Data are mean value ± SE. Bars with same letters are not significant at *p* ≤ 0.05 level. FC-0, FC-20, FC-40, FC-60, FC-80, and FC-100 are the water levels at 0, 20, 40, 60, 80, and 100% of field capacity, respectively

Gene expression analysis of CYP71D178 and CYP71D180 showed that drought stress promotes the carvacrol biosynthesis pathway in *P. amboinicus* (Fig. 5). Both genes were significantly upregulated under drought conditions, presenting an inverse relation with water availability. Specifically, the FC-0 treatment induced maximum expression levels (3.5-fold for *CYP71D178* and 4.9-fold for *CYP71D180*). In contrast, the 80% FC treatment resulted in the lowest expression gains, with transcripts only reaching 0.20 and 0.23 times the control values.

## Discussion

Drought stress is a major global hazard that impacts agricultural productivity, occurring due to high temperatures or low available water. Plant physiology is significantly impacted by the reduction in soil water, which causes adaptable species to respond to the osmotic effects of drought (Atteya et al. 2022). Under water stress conditions, plants undergoing phenotypic, morphological, and biochemical alterations for minimizing the moisture loss. During the vegetative phase, two important metrics used for evaluating a plant’s reaction to osmotic stress are growth and water status (Attia et al. 2022; Ghanem et al. 2022; Sheta et al. 2024a). In this study, water level impeded to *P. amboinicus* growth, where plants exposed to severe water deficiency (FC-0 and FC-20) exhibited the lowest shoot growth (Figs. 1 and 2). The decrease in shoot growth limits overall production, even though it helps restrict transpiration and prevent dehydration (Skirycz and Inzé 2010; Ngumbi and Kloepper 2016; El-Nagar et al. 2025). The reduction in shoot growth is a natural consequence of the essential role of water in supplying plant cells with the turgidity necessary for development (division, elongation, and expansion). Moreover, water is the medium for all cellular functions and vital processes. Such observation was reported by Sheta et al. (2024b), who revealed that decreasing available water levels were followed by a decrease in productivity.

Under drought stress, the reduction in vegetative growth leads to decrease in leaf biomass (size and area), which subsequently depresses overall biomass accumulation (Hessini et al. 2009). In *P. amboinicus*, drought had no significant effect on number of leaves, hence the observed reduction in herbaceous biomass was primarily driven by depression in leaf size and area. Sheta et al. (2024a) stated that a reduction in the biomass yield under drought stress reflects in photosynthetic activities, due to a decline in the photosynthetic area, lower pigment content, and stomatal closure. Notably, whilst there was a significant decrease in *P. amboinicus* growth under the lowest water levels, a substantial growth in essential oil production was observed, especially in the FC-0 treatment. Similar observations were previously reported by Farahanil et al. (2009); Chaves et al. 2011; Bettaieb et al. (2009) who noticed a reduction in the essential oil percentage of many aromatic plants cultivated under drought stress conditions. Petropoulos et al. (2008) stated that parsley plants grown under drought stress exhibited an increase in essential oil production, along with a reduction in biomass yield.

Photosynthetic pigments in *P. amboinicus* leaves showed a significant decline under severe and moderate water deficits (Table 4), while the highest chlorophyll and carotenoids concentrations were noticed in the FC-80 treatment. Leaf pigments are a good indicator for evaluating plant health under stressful conditions (Bahgat et al. 2023). Chlorophyll degradation and pigment photo-oxidation are the main reasons for the depression in photosynthetic pigment levels under water stress (Khalilzadeh et al. 2016; Gururani et al. (2023); ; ; ;. Carotenoids activate the antioxidant defense system in water-stressed plants by acting as antioxidants against different types of ROS. They enable plants to resist water stress by averting photo-oxidative deterioration in photosystem II (Sheta et al. 2024b). As non-enzymatic antioxidants, they have an important role in protecting membrane integrity from oxidative stress caused by reactive oxygen species. *P. amboinicus* plants exposed to severe drought conditions exhibited the lowest carotene content. The plant species, as well as the length and severity of water deficiency, affect the production of carotenoids (Nasrin et al. 2020). Numerous plants have shown signs of declining photosynthesis, metabolite production, and chlorophyll content under drought stress (Embiale et al. 2016; Husen et al. 2017).

Under water stress treatments, RWC and MSI of *P. amboinicus* leaves declined gradually with decreasing water level (Fig. 3). Drought stress negatively impacts the osmotic pressure in plant cells and, consequently, reduces the amount of water absorbed by roots. Under drought, plants suffer from low transpiration rates and poor water status. Every metabolic function that takes place in plant cells is heavily dependent on water (García et al. 2017). Reduced turgor pressure, caused by a drop in water levels, results in cell damage, slower plant growth, and general wilting. In contrast, high relative water content affects a plant’s capacity for recovery from stress and productivity (Lugojan and Ciulca 2011). Additionally, higher RWC aids in preventing osmotic stress and the accumulation of reactive oxygen species brought on by droughts leading to improved growth and production (Ngumbi and Kloepper 2016). Membrane degradation is one of the most common events evident in plant cells during environmental stress (El-Serafy et al. 2023). In the current study, MSI levels decreased with decreasing irrigationlevels. Under water stress conditions, oxidative stress affects cell membranes by oxidizing membrane lipids (Yordanov et al. 2000).

The unavoidable response of a plant to drought stress is elevated ROS generation. Over-accumulation of ROS can lead to many consequences, such as lipid peroxidation, peptide chain fragmentation, nucleotide elimination, and cell death (El-Serafy et al. 2024; Alayafi et al. 2025; Sharma et al. 2012; Elhakem and El-Serafy 2026). The final byproduct of lipid peroxidation is malondialdehyde, which indicate cellular damage (Miao et al. 2010; Chiappero et al. 2019; Kumar et al. 2019). *P. amboinicus* plants exposed to severe and moderate drought stress exhibited higher H_2_O_2_ and MDA levels, while FC-80 and FC-100 treatments exhibited a significant decline in these markers (Table 6). H_2_O_2_ has a harmful influence on plant cells, causing membrane lipid peroxidation, which was reflected in the accumulation of MDA (Guo et al. 2019). In order to control the generated reactive oxygen species, plants protect their cells from oxidative damage by generating non-enzymatic (e.g., β-carotenes and polyphenols) and enzymatic antioxidant systems (CAT and SOD) (Mittler 2002). CAT and SOD enzymes play a role in converting superoxide and hydrogen peroxide, leading to a reduction in the ROS buildup (El-Serafy et al. 2021; Mahdy et al. 2024; Al-Saif et al. 2024). These previous findings supported our results, which exhibited a significant increase in CAT and SOD levels in *P. amboinicus* leaves under the lowest water levels (Table 5). Furthermore, a notable increase in total polyphenol was noticed in leaves exposed to the lowest water levels. The results obtained revealed that antioxidant activity significantly decreased with increasing the water levels, proving that 80 and 100% of FC are the optimal levels for *P. amboinicus* growth.

Carvacrol, p-mentha-1, β-cymene, α-terpinolene, α-amorphene, beta-cubebene, à-caryophyllene, and α-caryophyllene are the major compounds detected in the *P. amboinicu*s essential oil (Table 5). Carvacrol, the main component of *P. amboinicu*s essential oil, is a phenol monoterpen, produced by the methy erythritol phosphate (MEP) pathway (Dudareva et al. 2005). The CYP71D178 and CYP71D180 genes have a critical role in carvacrol biosynthesis, as they catalyze the conversion of γ-terpinene to carvacrol (Crocoll et al. 2010; Kianersi et al. 2021). Our results showed that exhibited an increase in the transcription levels of CYP71D178 and CYP71D180 genes, accompanied by an increase in the carvacrol concentration; FC-0 plants showed the maximum levels (Fig. 5). Palesh and Abdollahi Mandoulakani (2020) discovered that in basil, genes responsible for monoterpene production are up-regulated during drought. Also, in sage plants, a significant rise in total monoterpene content is associated with a shift in the expression of multiple monoterpene synthases (Radwan et al. 2017). *Salvia officinalis* grown under drought conditions revealed a significantly higher concentration of monoterpenes relative to well-watered plants (Nowak et al. 2016). Drought stress stimulates the glycyrrhizin concentration in *Glycyrrhiza glabr*a roots, with an increase in the levels of important enzymes of genes encoding in triterpenoid biosynthesis (Nasrollahi et al. 2014). Drought stress stimulates the production of methyl chavicol and methyl eugenol in basil, along with an up-regulation of genes encoding the phenylpropanoid biosynthesis pathway (Abdollahi Mandoulakani et al. (2017). While Bahreininejad et al. (2013); Bahreininejad et al. (2014); Ghasemi Pirbalouti et al. (2014) reports suggest that drought decreases carvacrol content in other species, our finding suggests a strong positive correlation between water deficit and carvacrol synthesis in *P. amboinicus*.

Gama-terpinene and *p*-cymene are intermediate compounds in the carvacrol pathway. GC–MS analysis revealed an increase in their concentrations in stressed plants, and their levels were reduced with increasing water levels. In stressed plants, terpene concentrations are increased (Kesselmeier and Staudt 1999), which may indicate their role as radical scavengers (Grace and Logan 2000). Several phenolic compounds showed a significant increase in plants under water limitations. Generally, plants grown under drought stress accumulate higher levels of specialized metabolites relative to well-watered plants. These results demonstrated that the FC-0 treatment can be considered the optimal level to induce expression changes and stimulate the phytochemical accumulation. In other words, our findings indicate that drought stress may significantly increase carvacrol proportion in *P. amboinicus* and that the gene expression patterns of carvacrol biosynthesis-related genes are impacted by drought stress. In contrast to previous descriptive reports on *P. amboinicus*, this study discloses the genetic basis for chemotypic variation under different water levels. The identification of *CYP71D178* and *CYP71D180* as drought-inducible regulators provides a novel objective to enhance the pharmacological value of this species through strategic water management.

## Conclusion and future perspective

In conclusion, this report explains that while extreme water deficiency inhibits the vegetative growth of *P. amboinicus*, it markedly improves its chemical value by promoting the carvacrol biosynthetic pathway. The identification of the CYP71D genes as drought-responsive regulators provides a novel molecular objective for future biotechnological interventions. Broader implementations of these results involve the application of regulated deficit irrigation in farming for producing high-potency essential oils with minimize water consumption. Future studies should focus on overexpressing CYP71D178/180 genes, potentially allowing for high carvacrol production without biomass loss.

## Data Availability

All data generated or analyzed during this study are included in this published article.

## References

[CR1] Abdollahi MB, Eyvazpour E, Ghadimzadeh M (2017) The effect of drought stress on the expression of key genes involved in the biosynthesis of phenylpropanoids and essential oil components in basil (*Ocimum basilicum* L.). Phytochem 139:1–7 10.1016/j.phytochem.2017.03.006 28366608

[CR2] Aebi H (1984) Catalase in vitro. Methods Enzymol 105:121–126 6727660 10.1016/s0076-6879(84)05016-3

[CR3] Alayafi AH, Dahab AA, El-Sheshtawy ANA, Sharma A, Elhakem A, Youssef SM, El-Serafy RS (2025) Stimulatory effect of *Delonix regia f*lower extract in protecting *Syzygium cumini s*eedlings from salinity. Plants 14:875 40265752 10.3390/plants14060875 PMC11946084

[CR4] Al-Saif AM, Ahmed MEM, Taha MA, Sharma A, El-Sheshtawy ANA, Abouelsaad IA, El-Serafy RS, Mahdy RM (2024) Preharvest applications improve the postharvest storage and quality of Tomato fruits by enhancing the nutritional value and antioxidant system. Horticulturae 10:1248

[CR5] Alshallash KS, Mohamed MF, Dahab AA, Abd El-Salam HS, El-Serafy RS (2022) Biostimulation of *Plectranthus amboinicus* (Lour.) Spreng. with different yeast strains: morphological performance, productivity, phenotypic plasticity, and antioxidant activity. Horticulturae 8(10):887

[CR6] Arumugam G, Swamy MK, Sinniah UR (2016) *Plectranthus amboinicus* (Lour.) Spreng: botanical, phytochemical, pharmacological and nutritional significance. Molecules 21(4):369 27043511 10.3390/molecules21040369 PMC6274163

[CR7] Atteya AKG, El-Serafy RS, El-Zabalawy KM, Elhakem A, Genaidy EAE (2022) Exogenously supplemented proline and phenylalanine improve growth, productivity, and oil composition of salted moringa by up-regulating osmoprotectants and stimulating antioxidant machinery. Plants 11:1553 35736704 10.3390/plants11121553 PMC9227737

[CR8] Attia MA, Abou El-Enin MM, Abou Tahoun AM, Abdelghany FIM, El-Serafy RS (2022) Productivity of some barley cultivars as affected by supplemental irrigation under rainfed conditions. Aust J Crop Sci 16(5):665–675

[CR9] Bahgat AR, Dahab AA, Elhakem A, Gururani MA, El-Serafy RS (2023) Integrated action of rhizobacteria with *Aloe vera* and moringa leaf extracts improves defense mechanisms in *Hibiscus sabdariffa* L. cultivated in saline soil. Plants 12(21):3684 37960041 10.3390/plants12213684 PMC10648473

[CR10] Bahreininejad B, Razmjoo J, Mirza M (2013) Influence of water stress on morpho-physiological and phytochemical traits in *Thymus daenensis*. Int J Plant Product 7:151–166

[CR11] Bahreininejad B, Razmjoo J, Mirza M (2014) Effect of water stress on productivity and essential oil content and composition of *Thymus carmanicus*. J Essent Oil Bear Plants 17:717–725

[CR12] Barrs HD (1968) Determination of water deficits in plant tissues. In: Kozlowski TT (ed) Water deficits and plant growth, vol 1. Academic Press, New York, pp 235–368

[CR13] Beadle CL, Ludlow MM, Honeysett JL (1985) Chapter 5 - water relations. In: Coombs J, Hall DO, Long SP, Scurlock JMO (eds) Techniques in Bioproductivity and Photosynthesis, 2nd edn. Pergamon, pp 50–61

[CR14] Benzie IFF, Strain JJ (1996) The ferric reducing ability of plasma (FRAP) as a measure of “antioxidant power”: the FRAP assay. Anal Biochem 239:70–76 8660627 10.1006/abio.1996.0292

[CR15] Bettaieb I, Zakhama N, Aidi Wannes W, Kchouk ME, Marzouk B (2009) Water deficit effects on *Salvia officinalis* fatty acids and essential oils composition. Sci Hortic 120(2):271–275

[CR16] Bidabadi SS, VanderWeide J, Sabbatini P (2020) Exogenous melatonin improves glutathione content, redox state and increases essential oil production in two *Salvia* species under drought stress. Sci Rep 10(1):6883 32327687 10.1038/s41598-020-63986-6 PMC7181808

[CR17] Bouyoucos (1983) Les propriétés physiques du sol dépendent de sa texture et de sa structure. In: Les bases de la production végétale. Tome 1. Collection Sciences et Technique agricoles, pp. 67–87.

[CR19] Chaves MM, Costa JM, Saibo NJM (2011) Recent advances in photosynthesis under drought and salinity. Adv Bot Res 57:49–104

[CR20] Chiappero J, Cappellari LDR, Alderete LGS, Palermo TB, Banchio E (2019) Plant growth-promoting rhizobacteria improve the antioxidant status in *Mentha piperita* grown under drought stress leading to an enhancement of plant growth and total phenolic content. Ind Crops Prod 139:111553

[CR21] Crocoll C, Asbach J, Novak J, Gershenzon J, Degenhardt J (2010) Terpene synthases of oregano (*Origanum vulgare* L.) and their roles in the pathway and regulation of terpene biosynthesis. Plant Mol Biol 73:587–603 20419468 10.1007/s11103-010-9636-1

[CR22] de Abreu IN, Mazzafera P (2005) Effect of water and temperature stress on the content of active constituents of *Hypericum brasiliense* Choisy. Plant Physiol Biochem 43(3):241–248 15854832 10.1016/j.plaphy.2005.01.020

[CR23] Dewanto V, Wu X, Adom KK, Liu RH (2002) Thermal processing enhances the nutritional value of tomatoes by increasing total antioxidant activity. J Agric Food Chem 50:3010–3014 11982434 10.1021/jf0115589

[CR24] Dudareva N, Andersson S, Orlova I, Gatto N, Reichelt M, Rhodes D, Boland W, Gershenzon J (2005) The nonmevalonate pathway supports both monoterpene and sesquiterpene formation in snapdragon flowers. Proc Natl Acad Sci U S A 102:933–938 15630092 10.1073/pnas.0407360102 PMC545543

[CR25] Elhakem A, El-Serafy R (2026) The changes in growth and metabolic adaptation responses in java plum seedlings exposed to *Cassia javanica* extract under salinity. Plant Soil Environ. 10.17221/374/2025-pse

[CR26] El-Nagar AH, Ghanem KZ, Hassan FAS, Fetouh MI, El-Serafy RS, Moussa MM (2025) The changes in growth, yield, and biologically active compounds of essential oil in **Trachyspermum amm*i* L. upon rhizobacteria and seaweed applications. Plant Soil Environ 71:565–580

[CR27] El-Serafy RS, El-Sheshtawy ANA, Atteya AK, Al-Hashimi A, Abbasi AM, Al-Ashkar I (2021) Seed priming with silicon as a potential to increase salt stress tolerance in *Lathyrus odoratus*. Plants 10:2140. 10.3390/plants10102140 34685950 10.3390/plants10102140 PMC8539537

[CR28] El-Serafy RS, El-Sheshtawy ANA, Dahab AA (2023) Fruit peel soil supplementation induces physiological and biochemical tolerance in **Schefflera arboricol*a* L. grown under heat conditions. J Soil Sci Plant Nutr 23:1046–1059

[CR29] El-Serafy RS, Dahab AA, Ghanem KZ, Elhakem A, Bahgat AR, Venkatesh J, El-Sheshtawy AA, Badawy A (2024) As a natural antioxidant: *Sesbania grandiflora* leaf extract enhanced growth and yield performance, active ingredients and tolerance of *Hibiscus sabdariffa* L. under salt-affected soil. J Soil Sci Plant Nutr 24:3406–3420

[CR30] Elyasi R, Majdi M, Bahramnejad B, Mirzaghaderi G (2016) Spatial modulation and abiotic elicitors responses of the biosynthesis related genes of mono/triterpenes in black cumin (**Nigella sativ*a*). Ind Crops Prod 79:240–247

[CR31] Emami Bistgani Z, Barker AV, Hashemi M (2024) Physiology of medicinal and aromatic plants under drought stress. Crop J 12:330–339

[CR32] Embiale A, Hussein M, Husen A, Sahile S, Mohammed K (2016) Differential sensitivity of *Pisum sativum* L. cultivars to water-deficit stress: changes in growth, water status, chlorophyll fluorescence and gas exchange attributes. J Agron 15(2):45–57

[CR33] Erny SMN, Razali M, Mirfat AHS, Mohd Shukri MA (2014) Antimicrobial activity and bioactive evaluation of **Plectranthus amboinicu*s* essential oil. Am J Res Commun 2(12):121–127

[CR34] Farahani HA, Valadabadi SA, Daneshian J, Khalvati MA (2009) Evaluation changing of essential oil of balm (*Melissa officinalis* L.) under water deficit stress conditions. J Med Plant Res 3(5):329–333

[CR35] García JE, Maroniche G, Creus C, Suárez-Rodríguez R, Ramirez Trujillo JA, Groppa MD (2017) *In vitro* PGPR properties and osmotic tolerance of different **Azospirillu*m* native strains and their effects on growth of maize under drought stress. Microbiol Res 202:21–29 28647119 10.1016/j.micres.2017.04.007

[CR37] Ghanem KZ, Hasham MMA, El-Sheshtawy AA, El-Serafy RS, Sheta MH (2022) Biochar stimulated actual evapotranspiration and wheat productivity under water deficit conditions in sandy soil based on non-weighing lysimeter. Plants Basel 11(23):3346 36501385 10.3390/plants11233346 PMC9735446

[CR38] Ghasemi Pirbalouti A, Rahmani Samani M, Hashemi M, Zeinali H (2014) Salicylic acid affects growth, essential oil and chemical compositions of thyme (**Thymus daenensi*s* Celak.) under reduced irrigation. Plant Growth Regul 72:289–301

[CR39] Giannopolitis CN, Ries SK (1977) Superoxide dismutases: I. occurrence in higher plants. Plant Physiol 59(2):309–314 16659839 10.1104/pp.59.2.309 PMC542387

[CR40] Grace SC, Logan BA (2000) Energy dissipation and radical scavenging by the plant phenylpropanoid pathway. Philos Trans R Soc Lond B Biol Sci 355:1499–1510 11128003 10.1098/rstb.2000.0710 PMC1692864

[CR41] Grayer RJ, Eckert MR, Lever A, Veitch NC, Kite GC, Paton AJ (2010) Distribution of exudate flavonoids in the genus *Plectranthus*. Biochem Syst Ecol 38(3):335–341

[CR42] Guo Q, Liu L, Barkla BJ (2019) Membrane lipid remodeling in response to salinity. Int J Mol Sci 20:4264 31480391 10.3390/ijms20174264 PMC6747501

[CR43] Gururani MA, Atteya AK, Elhakem A, El-Sheshtawy AA, El-Serafy RS (2023) Essential oils prolonged the cut carnation longevity by limiting the xylem blockage and enhancing the physiological and biochemical levels. PLoS ONE 18(3):e0281717 36881583 10.1371/journal.pone.0281717 PMC9990951

[CR44] Heath RL, Packer L (1968) Photoperoxidation in isolated chloroplasts. I. Kinetics and stoichiometry of fatty acid peroxidation. Arch Biochem Biophys 125(1):189–198 5655425 10.1016/0003-9861(68)90654-1

[CR45] Hessini K, Martínez JP, Gandour M, Albouchi A, Soltani A, Abdelly C (2009) Effect of water stress on growth, osmotic adjustment, cell wall elasticity and water-use efficiency in *Spartina alterniflora*. Environ Exp Bot 67(2):312–319

[CR46] Holm G (1954) Chlorophyll mutations in barley. Acta Agric Scand 4:457–471

[CR47] Husen A, Iqbal M, Aref IM (2017) Plant growth and foliar characteristics of faba bean (*Vicia faba* L.) as affected by indole-acetic acid under water-sufficient and water-deficient conditions. J Environ Biol 38(2):179–186

[CR48] Kesselmeier J, Staudt M (1999) Biogenic volatile organic compounds (VOC): An overview on emission, physiology and ecology. J Atmos Chem 33:23–88

[CR49] Khalilzadeh R, Sharifi RS, Jalilian J (2016) Antioxidant status and physiological responses of wheat (**Triticum aestivu*m* L.) to cycocel application and bio fertilizers under water limitation condition. J Plant Interact 11(1):130–137

[CR50] Khodabin G, Lightburn K, Hashemi SM, Moghada MSK, Jalilian A (2022) Evaluation of nitrate leaching, fatty acids, physiological traits and yield of rapeseed (*Brassica napus*) in response to tillage, irrigation and fertilizer management. Plant Soil 473:423–440

[CR51] Kianersi F, PourAboughadareh A, Majdi M, Poczai P (2021) Effect of methyl jasmonate on thymol, carvacrol, phytochemical accumulation, and expression of key genes involved in thymol/carvacrol biosynthetic pathway in some Iranian thyme species. Int J Mol Sci 22:11124. 10.3390/ijms222011124 34681782 10.3390/ijms222011124 PMC8539593

[CR52] Kleinwächter M, Selmar D (2015) New insights explain that drought stress enhances the quality of spice and medicinal plants: Potential applications. Agron Sustain Dev 35:121–131

[CR53] Kumar A, Patel JS, Meena VS, Srivastava R (2019) Recent advances of PGPR based approaches for stress tolerance in plants for sustainable agriculture. Biocatal Agric Biotechnol 20:101271

[CR54] Latif M, Bukhari SAH, Alrajhi AA, Alotaibi FS, Ahmad M, Shahzad AN, Dewidar AZ, Mattar MA (2022) Inducing drought tolerance in wheat through exopolysaccharide-producing *rhizobacteria*. Agronomy 12:1140

[CR55] Livak KJ, Schmittgen TD (2001) Analysis of relative gene expression data using real-time quantitative PCR and the 2−∆∆ct method. Methods 25:402–408 11846609 10.1006/meth.2001.1262

[CR56] Lugojan C, Ciulca S (2011) Evaluation of relative water content in winter wheat. J Hortic Sci Biotechnol 15(2):173–177

[CR57] Mahdy RM, Al-Saif AM, Ahmed MEM, El-Bary TSA, Sharma A, El-Sheshtawy ANA, El-Serafy RS, El-Ghany TSA (2024) Evaluation of two different methods of fulvic acid application (seed priming and foliar spray) on growth, yield, and nutritional quality of pea (**Pisum sativu*m* L.). Plants Basel 13:3380 39683173 10.3390/plants13233380 PMC11644410

[CR58] Miao BH, Han XG, Zhang WH (2010) The ameliorative effect of silicon on soybean seedlings grown in potassium-deficient medium. Ann Bot 105(6):967–973 20338952 10.1093/aob/mcq063 PMC2876006

[CR59] Mitic V, Jovanovic VS, Dimitrijevic M, Cvetkovic J, Stojanovic G (2013) Effect of food preparation technique on antioxidant activity and plant pigment content in some vegetables species. J Food Nutr Res 1:121–127

[CR60] Mittler R (2002) Oxidative stress, antioxidants and stress tolerance. Trends Plant Sci 7(9):405–410 12234732 10.1016/s1360-1385(02)02312-9

[CR61] Muniandy K, Hassan Z, Isa MHM (2014) The action of *Coleus aromaticus* as a potential wound healing agent in experimentally induced diabetic mice. PERINTIS E-J 4(1):1–30

[CR62] Murthy PS, Ramalakshmi K, Srinivas P (2009) Fungitoxic activity of Indian borage (*Plectranthus amboinicus*) volatiles. Food Chem 114(3):1014–1018

[CR63] Nasrin S, Saha S, Begum HH, Samad R (2020) Impacts of drought stress on growth, protein, proline, pigment content and antioxidant enzyme activities in rice (**Oryza sativ*a* L. var. BRRI dhan-24). Dhaka Univ J Biol Sci 29(1):117–123

[CR64] Nasrollahi V, Mirzaie-asl A, Piri K, Nazeri S, Mehrabi R (2014) The effect of drought stress on the expression of key genes involved in the biosynthesis of triterpenoid saponins in liquorice (*Glycyrrhiza glabra*). Phytochemistry 103:32–37 24768283 10.1016/j.phytochem.2014.03.004 PMC7111703

[CR65] Ngumbi E, Kloepper J (2016) Bacterial-mediated drought tolerance: current and future prospects. Appl Soil Ecol 105:109–125

[CR66] Nowak M, Selmar D (2016) Cellular distribution of alkaloids and their translocation via phloem and xylem: the importance of compartment pH. Plant Biol 18:879–882 27606889 10.1111/plb.12504

[CR67] Palesh H, Abdollahi-Mandoulakani B (2020) The effect of drought stress on the expression of some genes involved in monoterpene and sesquiterpanes biosynthesis and essential oil compounds in basil. J Med Plants 19:204–212

[CR68] Patterson BD, MacRae EA, Ferguson IB (1984) Estimation of hydrogen peroxide in plant extracts using titanium (IV). Anal Biochem 139(2):487–492 6476384 10.1016/0003-2697(84)90039-3

[CR69] Petropoulos SA, Daferera D, Polissiou MG, Passam HC (2008) The effect of water deficit stress on the growth, yield and composition of essential oils of parsley. Sci Hortic 115(4):393–397

[CR70] Pradhan J, Sahoo SK, Lalotra S, Sarma RS (2017) Positive impact of abiotic stress on medicinal and aromatic plants. Int J Plant Sci 12(2):309–313

[CR71] Radwan A, Kleinwächter M, Selmar D (2017) Impact of drought stress on secondary metabolism: biosynthesis and the expression of monoterpene synthases in sage (*Salvia officinalis*). Phytochemistry 141:20–26 28550743 10.1016/j.phytochem.2017.05.005

[CR72] Ramezani S, Abbasi A, Sobhanverdi S, Shojaeiyan A, Ahmadi N (2020) The effects of water deficit on the expression of monoterpene synthases and essential oils composition in *Salvia* ecotypes. Physiol Mol Biol Plants 26:2199–2207. 10.1007/s12298-020-00892-1 33268923 10.1007/s12298-020-00892-1 PMC7688846

[CR73] Roshan P, Naveen M, Manjul PS, Gulzar A, Anita S, Sudarshan S (2010) *Plectranthus amboinicus* (Lour) Spreng: an overview. Pharm Res 4:1–15

[CR74] Sairam RK, Deshmukh PS, Shukla DS (1997) Tolerance to drought and temperature stress in relation to increased antioxidant enzyme activity in wheat. J Agron Crop Sci 178:171–178. 10.1111/j.1439-037x.1997.tb00486.x

[CR75] Selmar D, Kleinwächter M (2013) Influencing the product quality by deliberately applying drought stress during the cultivation of medicinal plants. Ind Crops Prod 42:558–566

[CR76] Sharma P, Jha A, Dubey RS, Pessarakli M (2012) Reactive oxygen species, oxidative damage, and antioxidative defence mechanism in plants under stressful conditions. J Bot 2012:217037

[CR77] Sheta MH, Hasham MMA, Ghanem KZ, Bayomy HM, El-Sheshtawy AA, El-Serafy RS, Naif E (2024a) Screening of wheat genotypes for water stress tolerance using soil–water relationships and multivariate statistical approaches. Agronomy (Basel) 14(5):1029

[CR78] Sheta MH, El-wahed AHMA, Elshaer MAA, Bayomy HM, Ozaybi NA, Abd-Elraheem MAM, El-Sheshtawy ANA, El-Serafy RS, Moustafa MMI (2024b) Green synthesis of zinc and iron nanoparticles using **Psidium guajav*a* leaf extract stimulates cowpea growth, yield, and tolerance to saline water irrigation. Horticulturae (Basel) 10:915. 10.3390/horticulturae10090915

[CR79] Singh-Sangwan N, Abad Farooqi AH, Sangwan RS (1994) Effect of drought stress on growth and essential oil metabolism in lemongrasses. New Phytol 128(1):173–179 33874545 10.1111/j.1469-8137.1994.tb04000.x

[CR80] Skirycz A, Inzé D (2010) More from less: plant growth under limited water. Curr Opin Biotechnol 21(2):197–203 20363612 10.1016/j.copbio.2010.03.002

[CR82] Soni U, Brar S, Gauttam VK (2015) Effect of seasonal variation on secondary metabolites of medicinal plants. Int J Pharm Sci Res 6(9):3654–3662

[CR83] Tohidi B, Rahimmalek M, Arzani A, Trindade H (2020) Sequencing and variation of terpene synthase gene (TPS2) as the major gene in biosynthesis of thymol in different *Thymus* species. Phytochemistry 1:112–126 10.1016/j.phytochem.2019.112126 31644985

[CR84] Valera D, Rivas R, Avila JL, Aubert L, Amelot MA, Usubillaga A (2003) The essential oil of *Coleus amboinicus* Loureiro, chemical composition and evaluation of insect anti-feedant effects. Ciencia 11(2):113–118

[CR85] Viuda-Martos M, Mohamady MA, Fernández-López J, AbdElRazik KA, Omer EA, Pérez-Alvarez JA, Sendra E (2011) In vitro antioxidant and antibacterial activities of essentials oils obtained from Egyptian aromatic plants. Food Control 22(11):1715–1722

[CR86] Von Wettstein D (1957) Chlorophyll-letale und der submikroskopische formwechsel der plastiden. Exp Cell Res 12:427–506 13437976 10.1016/0014-4827(57)90165-9

[CR87] Winter K, Virgo A, Garcia M, Aranda J, Holtum JA (2020) Constitutive and facultative crassulacean acid metabolism (CAM) in *Cuban oregano*, *Coleus amboinicus* (Lamiaceae). Funct Plant Biol 48(7):647–654 10.1071/FP20127 32919492

[CR89] Yordanov I, Velikova V, Tsonev T (2000) Plant responses to drought, acclimation, and stress tolerance. Photosynthetica 38(2):171–186

[CR90] Youssef SM, El-Serafy RS, Ghanem KZ, Elhakem A, Abdel Aal AA (2022) Foliar spray or soil drench: microalgae application impacts on soil microbiology, morpho-physiological and biochemical responses, oil and fatty acid profiles of chia plants under alkaline stress. Biology Basel 11(2):1844 36552353 10.3390/biology11121844 PMC9775337

